# SETD8 cooperates with MZF1 to participate in hyperglycemia-induced endothelial inflammation via elevation of WNT5A levels in diabetic nephropathy

**DOI:** 10.1186/s11658-022-00328-6

**Published:** 2022-03-26

**Authors:** Fei Wang, Wenting Hou, Xue Li, Lihong Lu, Ting Huang, Minmin Zhu, Changhong Miao

**Affiliations:** 1 Department of Anesthesiology, Fudan University Shanghai Cancer Center; Department of Oncology, Shanghai Medical College, Fudan University, Shanghai, 200032 China; 2 Department of Anesthesiology, Shanghai General Hospital, Shanghai Jiao Tong University School of Medicine, Shanghai, 200080 People’s Republic of China; 3 Department of Anesthesiology, Zhongshan Hospital, Fudan University, Shanghai, 200032 China

**Keywords:** Diabetic nephropathy, SETD8, Endothelial

## Abstract

**Objective:**

Diabetic nephropathy (DN) is regarded as the main vascular complication of diabetes mellitus, directly affecting the outcome of diabetic patients. Inflammatory factors were reported to participate in the progress of DN. Wingless-type family member 5 (WNT5A), myeloid zinc finger 1 (MZF1), and lysine methyltransferase 8 (SETD8) have also been reported to elevate inflammatory factor levels and activate the nuclear factor kappa B (NF-κB) pathway to induce endothelial dysfunction. In the current study, it was assumed that MZF1 associates with SETD8 to regulate WNT5A transcription, thus resulting in hyperglycemia-induced glomerular endothelial inflammation in DN.

**Methods:**

The present study recruited 25 diagnosed DN patients (type 2 diabetes) and 25 control participants (nondiabetic renal cancer patients with normal renal function, stage I–II) consecutively. Moreover, a DN rat and cellular model was constructed in the present study. Immunohistochemistry, Western blot, and quantitative polymerase chain reaction (qPCR) were implemented to determine protein and messenger RNA (mRNA) levels. Coimmunoprecipitation (CoIP) and immunofluorescence were implemented in human glomerular endothelial cells (HGECs). Chromatin immunoprecipitation assays and dual luciferase assays were implemented to determine transcriptional activity.

**Results:**

The results of this study indicated that levels of WNT5A expression, p65 phosphorylation (p-p65), and inflammatory factors were all elevated in DN patients and rats. In vitro, levels of p-p65 and inflammatory factors increased along with the increase of WNT5A expression in hyperglycemic HGECs. Moreover, high glucose increased MZF1 expression and decreased SETD8 expression. MZF1 and SETD8 inhibit each other under the stimulus of high glucose, but cooperate to regulate WNT5A expression, thus influencing p-p65 and endothelial inflammatory factors levels. Overexpression of MZF1 and silencing of SETD8 induced endothelial p-p65 and inflammatory factors levels, which can be reversed by si-WNT5A. Mechanistic research indicated that MZF1, SETD8, and its downstream target histone H4 lysine 20 methylation (H4K20me1) all occupied the WNT5A promoter region. sh-SETD8 expanded the enrichment of MZF1 on WNT5A promoter. Our in vivo study proved that SETD8 overexpression inhibited levels of WNT5A, p-p65 expression, and inflammatory factors in DN rats.

**Conclusions:**

MZF1 links with SETD8 to regulate WNT5A expression in HGECs, thus elevating levels of hyperglycemia-mediated inflammatory factors in glomerular endothelium of DN patients and rats.

*Trial registration* ChiCTR, ChiCTR2000029425. 2020/1/31, http://www.chictr.org.cn/showproj.aspx?proj=48548

**Supplementary Information:**

The online version contains supplementary material available at 10.1186/s11658-022-00328-6.

## Background

Diabetic nephropathy (DN), which can result in serious health problems and great financial burden to human society, is one of the main microvascular complications induced by diabetes mellitus (DM) [1]. In developed countries, DN occurs in about 20–40% of type 2 DM patients and accounts for around 50% of cases, while in China, the incidence and prevalence of DN have also increased dramatically over the past decade [2, 3]. However, current management of DN, such as blocking the renin–angiotensin–aldosterone system, blood glucose level control, and body weight reduction, fail to achieve satisfactory outcomes [4]. As a result, it is urgent to develop new and effective therapeutic approaches to prevent the genesis and development of DN, which requires full understanding of the mechanism of DN.

Although DN has not been considered to be an immune disease, inflammation (characterized by elevation of various inflammatory factors, including interleukin (IL)-6 and tumor necrosis factor-alpha (TNFα)) has been proved to participate in the pathogenesis of DN [5]. Serum TNFα levels are upregulated in diabetic patients, which enhances glomerular vasoconstriction and the filtration rate [6]. IL-6 has been reported to participate in inflammation responses central to the progression of DN [7]. Wnt family member 5A (WNT5A) is a novel mediator of inflammation in metabolic diseases and has been reported to be a typical ligand of noncanonical Wnt signaling [8–10]. WNT5A plays a novel role in inflammatory processes through NF-κB pathway modulation [11, 12]. By directly combining with CD146, WNT5A-mediated noncanonical signaling is a factor contributing to renal tubular inflammation in DN [13]. However, what regulates WNT5A levels in DN it is still not well known. It has been reported that epigenetic modification participates in the modulation of the WNT5A signaling pathway [14]. Moreover, epigenetic modification is believed to play an important role in the pathogenesis of diabetic nephropathy [15].

SETD8, the only lysine methyltransferase to be found to be involved in the monomethylation of lysine 20 of histone H4 (H4K20me1), plays a crucial role in DNA replication, cell cycle regulation, genomic instability, transcriptional modulation, cellular metabolism, etc. [16, 17]. Our previous study revealed that suppression of SETD8 participates in high-glucose-mediated vascular endothelial injury, thus mediating the development of diabetic nephropathy [18, 19]. SETD8 may thus possibly become a potential therapeutic target in diabetic nephropathy.

Myeloid zinc finger 1 (MZF1) serves as a transcription factor belonged to the Kruppel family of zinc fingers, which is preferentially expressed in myeloid progenitor cells [20]. It is mainly located in hematopoietic progenitors and cells of the myeloid lineage with a very narrow tissue distribution, and MZF1 plays an important role in cell growth, differentiation, and apoptosis [21]. In the current study, we assume that MZF1 cooperates with SETD8 to regulate WNT5A transcript, thus affecting inflammatory factors levels in DN.

## Methods

### Subjects

Our current study recruited 25 diagnosed DN patients (type 2 diabetes) and 25 control participants (nondiabetic renal cancer patients with normal renal function, stage I–II) consecutively. The study was authorized by the Ethics Committee of Huzhou Central Hospital (license no. 20191209-01) and complied with the Declaration of Helsinki. Written informed consent was acquired from all participants. Serum samples from subjects were kept at −80 °C until analysis. Serum IL-6 and TNFα levels were detected by using an IL-6 assay kit (Jiancheng Bio, Nanjing, China) and a TNFα assay kit (Jiancheng Bio, Nanjing, China), respectively.

### Rat model of DN and samples

Male Sprague–Dawley rats weighing about 180–200 g were obtained from Shanghai SLAC Laboratories. The present study complied with the Guide for the Care and Use of Laboratory Animals of Shanghai Medical College of Fudan University and was performed according to the Institutional Guidelines for Animal Research and the Guide for the Care and Use of Laboratory Animals published by the US NIH (2011). All rats received unilateral nephrectomy (Unx) under isoflurane anesthesia (3–4% induction, 1.5–2.5% maintenance, 100% oxygen). Three weeks after Unx, the rats receiving a single intraperitoneal injection of citrate buffer (0.1 M, pH 4.5) were defined as the control group (con, *n* = 10). Rats treated with a high-sugar/fat diet for 9 weeks and intraperitoneal injection of streptozotocin (STZ, 50 mg/kg) 3 weeks after Unx were defined as the DN group (DN, *n* = 10). To reveal the protective effect of SETD8 overexpression against DN, DN rats were injected with AAV-SETD8 or control vectors into the contralateral kidney at the time of Unx. These rats were defined as the DN + AAV group (DN + AAV, *n* = 10) and DN + AAV-SETD8 group (DN + SETD8, *n* = 10). Three days after STZ injection, hyperglycemia was confirmed by measuring blood glucose via tail-neck blood sampling. In the present study, a successful DN model should meet two criteria: an increase in albuminuria levels by more than about sixfold, and an increase in blood glucose levels by more than 300 mg/dL. STZ-treated rats whose blood glucose levels were less than 300 mg/dL were excluded from the present study. The percentage of rats that developed hyperglycemia and DN was about 100% and 75%, respectively. The day before euthanasia, 24-h urine samples of the rats were gathered in metabolic cages. Blood samples of the rats were gathered in ethylenediaminetetraacetic acid (EDTA) vacutainer tubes. The samples were frozen at −80 °C until analysis. A creatinine (CREA) assay kit (Puruibo Bio, Ningbo, China) and a blood urea nitrogen (BUN) assay kit (Puruibo Bio, Ningbo, China) were employed to measure serum CREA and BUN in this study. An enhanced bicinchoninic acid (BCA) protein assay kit (Beyotime, Shanghai, China) was employed to determine urinary albumin.

### Masson staining and immunohistochemistry (IHC)

Masson staining was preformed to evaluate the severity of fibrosis in tissues according to the instructions (Solarbio, Beijing, China). Standard immunohistochemistry (IHC) procedures were performed by using anti-WNT5A (Sabbiotech, USA, #49719), anti-p-p65 (ProteinTech, Wuhan, China, 14063–1-AP), anti-MZF1 (Sabbiotech, USA, #33737), and anti-SETD8 (ProteinTech, Wuhan, China, 14063-1-AP) antibodies.

### Cell culture and reagents

Human glomerular endothelial cells (HGECs) were obtained from Procell (Wuhan, China). The cells cultured in Dulbecco’s modified Eagle’s medium (DMEM) with 5 mM glucose were defined as normal-glucose group. The cells cultured in DMEM with 25 mM glucose for 6 days were defined as high-glucose treatment. Glucose (5 mM) plus mannitol (20 mM) was used to eliminate the influence of osmotic pressure.

### Western blot analysis

After washing with cold phosphate-buffered saline (PBS), HGECs were lysed in radioimmunoprecipitation assay (RIPA) lysis buffer (Cell Signalling Technology, Danvers, MA) with phenylmethylsulfonyl fluoride (PMSF) (Beyotime Biotechnology, Shanghai). Protein concentration was determined by using a BCA protein assay kit (Epizyme, Shanghai, China). Equal amount of protein (50 μg) from each group was separated by sodium dodecyl sulfate–polyacrylamide gel electrophoresis and transferred to polyvinylidene fluoride (PVDF) membranes (Millipore, Billerica, USA). The primary antibodies used in the current study were as follows: monoclonal antibodies against β-actin (ProteinTech, Wuhan, China, 60004-1-Ig), SETD8 (ProteinTech, Wuhan, China, 14063–1-AP), WNT5A (Sabbiotech, USA, #49719), p-p65 (ProteinTech, Wuhan, China, 14063-1-AP), and MZF1 (Sabbiotech, USA, #33737).

### Quantitative real-time PCR (qPCR)

Total RNA from HGECs or tissues was obtained using TRIzol (Invitrogen, Grand Island, NY, USA), and complementary DNA (cDNA) was synthesized through reverse transcript with PrimeScript RT reagent (TaKaRa, Dalian, China). Hieff qPCR SYBR Green Master Mix (Yeasen, Shanghai, China) was used to perform real-time PCR in an ABI7500 real-time PCR system (Applied Biosystems). All transcript levels are referred to β-actin levels. The primers in the current study are presented in Additional file 1: Table S1.

### Bioinformatics analysis of underlying protein associations

The STRING (https://string-db.org/) database was employed to determine the underlying associations between specific proteins and unknown proteins. Potential proteins associated with SETD8 were sought by analyzing the STRING database.

### Coimmunoprecipitation (CoIP)

HGECs were plated in 100-mm dishes and cultured for about 48–60 h, when they were 90–100% complete. The cells were harvested and extracted with cell lysis buffer (Cell Signalling Technology, Danvers, MA) containing PMSF (Beyotime Biotechnology, Shanghai) to obtain protein lysates. Lysate supernatant (30 μL) was spared as the input. The other supernatant was incubated with the corresponding primary antibodies and A/G Dynabeads (Thermo Fisher, USA) at 4 °C overnight to obtain endogenous IP. Western blotting was preformed to analyze the input, IgG, and IP fractions.

### Immunofluorescence (IF) staining

HGECs were plated onto confocal dishes with the corresponding treatments 1 day in advance. HGECs were incubated with anti-SETD8 (Proteintech, Wuhan, China, 14,063–1-AP) and anti-MZF1 (Sabbiotech, USA, #33737) antibodies at 4 °C overnight. After washing thrice with PBS, corresponding florescent secondary antibodies (Solarbio, Beijing, China) were added for 1 h at room temperature. Finally, 4′,6-diamidino-2-phenylindole (DAPI) was employed for nuclear staining. Images were acquired by confocal fluorescence microscopy (Leica).

### Chromatin immunoprecipitation (ChIP) assay

ChIP experiments were performed using a Simple ChIP plus sonication chromatin IP kit (Cell Signalling Technology, MA). Briefly, HGECs (1 × 10^7^) were fixed with 1% formaldehyde for 10 min at room temperature to cross-link the DNA and the proteins, then terminated by adding glycine. The chromatin was sheared by using a Microson XL ultrasonic cell disruptor XL (Misonix). Ten microliters of sonicated solution was spared as the input. The remaining was incubated with anti-MZF1 (Sabbiotech, USA, #33737), anti-SETD8 (Pro-teinTech, Wuhan, China, 14063-1-AP), anti-H4K20me1 antibody (Abcam, Cambridge, UK, ab9051) and anti-Histone H3 (Abcam, Cambridge, UK, ab1791) or negative control IgG, respectively, at 4 °C overnight. After purification, the enriched DNA sequences were detected by PCR and agarose gel electrophoresis on a 2% agarose TAE gel. The WNT5A oligonucleotide primer sequences were as follows: prime 1 (P1): forward, 5′-TTTTGCGACACCTGTGCTTG-3′, and reverse 5′-TGCCTGTTCTGTCCACTCA-3′; prime 2 (P2): forward, 5′-TAGTACAAGAGAATGGACTA-3′, and reverse 5′-CACCAGCCCAGCTCACCTCA-3′; prime 3 (P3): forward, 5′-CCAGATTGTATATTTGATGC-3′, and reverse, 5′-AGATAGTAAGTGGTGGAGCC-3′; primer 4 (P4): forward, 5′-TACCAGGCTGACACACTGCT-3′, and reverse 5′-GAATTACTGATCTGTAATAA-3′; prime 5 (P5): forward, 5′-ACTGTCCTCAGAGCTCTTGA-3′, and reverse 5′-AGCCACCACATGTGACCTTC-3′.

### RNA interference and dual-luciferase assay

HGECs were transfected with sh-SETD8, si-MZF1, and si-WNT5A using Lipofectamine 3000 (Invitrogen, USA). The sequences of sh-SETD8 in this study (Biotend, Shanghai, China) were shRNA-a, 5′-CAACAGAATCGCAAACTTA-3′ and shRNA-b, 5′-CAACAGAATCGCAAACTTA-3′. The sequences of si-WNT5A (Biotend, Shanghai, China) were siRNA-a, 5′-GUGGUCGCUAGGUAUGAAU-3′, siRNA-b, 5′-GCUACGUCAAGUGCAAGAA-3′, and siRNA-c, 5′-GCCAGUAUCAAUUCCGACA-3′. The sequences of si-MZF1 (Biotend, Shanghai, China) were siRNA-a, 5′-GUGUCAUGGUGAAGCUAGA-3′ and siRNA-b, 5′-CGAGGAGGCUGCUGCCUA-3′.

The WNT5A promoter (−21 bp to −275 bp) was amplified and ligated into the pGL3 basic vector to create the pGL3-WNT5A construct. Meanwhile, the mutation of the promoter site (ATAATCCCCACAT-CGCCGAAAACACG) was created for comparison. HGECs were then transfected with the pGL3-WNT5A plasmid and pGL3-WNT5A^mut^ plasmid along with a *Renilla* luciferase vector. The Promega dual-luciferase assay Kit (Madison, WI, USA) was used to assess the effects of MZF1 on the activity of the WNT5A promoter.

### Statistical analysis

The sample size of animals and HGECs was confirmed by assessing hyperglycemia and high-glucose-induced WNT5A mRNA levels. Results are presented as mean ± standard deviation (SD) acquired from at least five experiments performed separately. Two-tailed unpaired *t* tests or one-way analyses of variance (ANOVAs) with GraphPad Prism version 8.0 (GraphPad Software, San Diego, CA) were used to compare groups. *p* < 0.05 was considered significant.

## Results

### Plasma inflammatory factors levels, p65 phosphorylation, and glomerular WNT5A levels were elevated in DN patients and rats

**Fig. 1:**
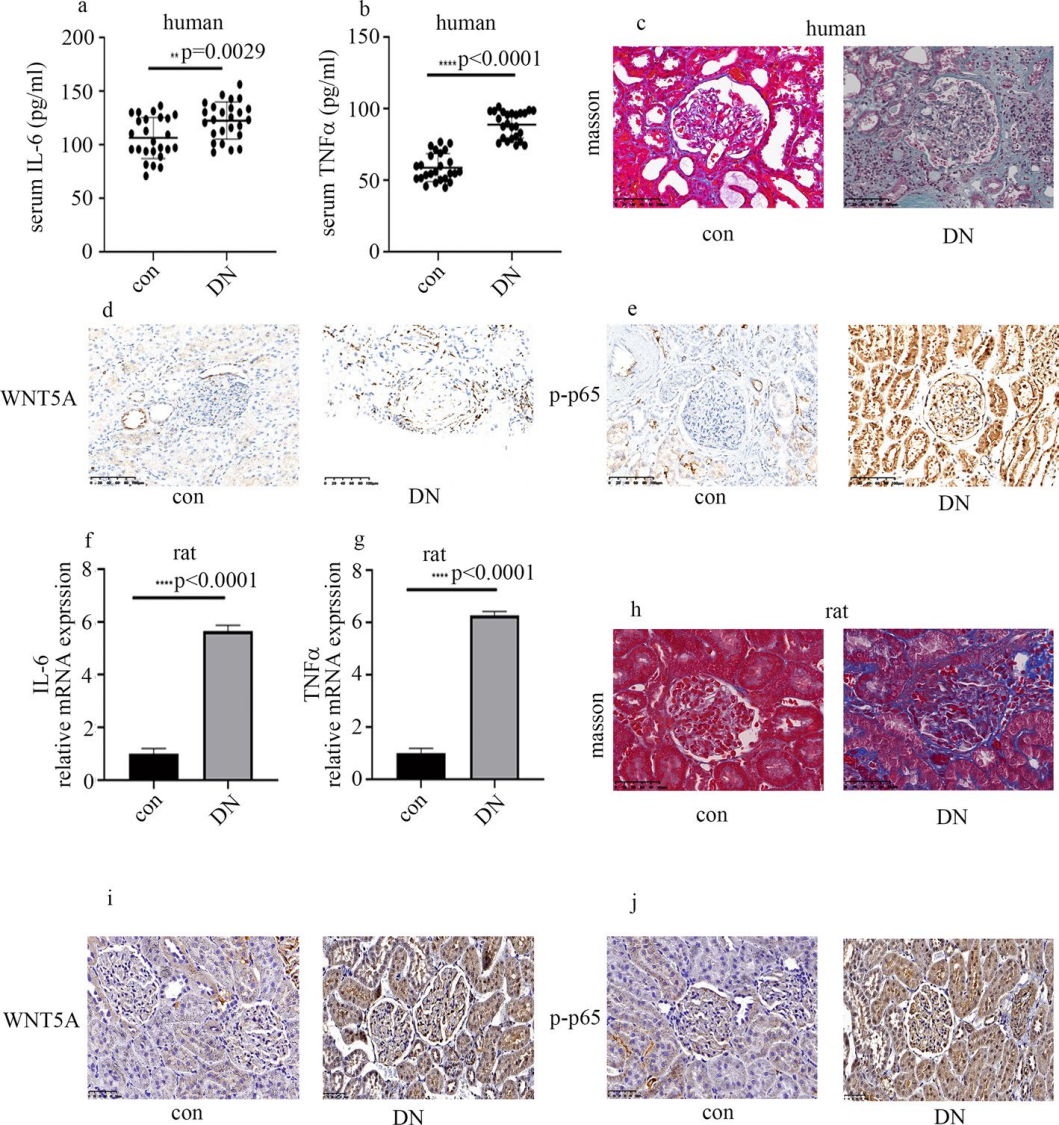
The degree of inflammation and fibrosis were increased in kidney of DN patients and rats. **a**, **b** Plasma IL-6 and TNFα in human. **c** Masson staining of human renal specimens. **d**, **e** IHC assay of WNT5A and p-p65 in kidney of participants. **f**, **g** mRNA expression of IL-6 and TNFα in kidney of rats. **h** Masson staining of rat renal specimens. **i**, **j** IHC assay of WNT5A and p-p65 in kidney of rats (*p < 0.05, **p < 0.01, ***p < 0.001, ****p < 0.0001, *n* = 10 per group)

To examine whether inflammatory factors were involved in DN, levels of plasma inflammatory factors were measured in participants. The characteristics of the participants in the current study are presented in Table 1. Plasma levels of IL-6 and TNFα were elevated in DN patients (Fig. 1a, b). With progress of inflammation in DN, fibroblasts are recruited, ultimately resulting in renal fibrosis and injury. Masson trichome staining of renal biopsy specimens was carried out (Fig. 1c). It has been reported that WNT5A regulates NF-κB activity [11]. Therefore, we detected WNT5A and p-p65 levels in renal specimens. The results showed that WNT5A and p-p65 expression in glomerular endothelial cells were both higher in DN than control participants (Fig. 1d, e). These data indicate that levels of plasma inflammatory factors, p-p65, and WNT5A in kidney tissues may participate in development of DN.

**Table 1:** Characteristics of participants and rats in control (con) and diabetic nephropathy (DN) groups

Variable	Con	DN	*p*-Value
*Human*			
Male (%)	53%	70%	0.7160
Age (years)	52.38 ± 15.06	53.82 ± 9.23	0.7475
BMI (kg/m^2^)	25.34 ± 2.96	24.61 ± 2.65	0.4789
SBP (mmHg)	140.8 ± 17.68	148.1 ± 23	0.3362
DBP (mmHg)	77.5 ± 8.94	80.56 ± 14.79	0.4978
FBG (mmol/L)	5.42 ± 1.19	10.43 ± 5.14	< 0.001
CREA (umol/L)	88.76 ± 32.45	193.7 ± 105.3	< 0.001
CCr (mL/min)	103.1 ± 31.04	54.75 ± 25.93	< 0.0001
24hUTP (mg)	353.8 ± 134.58	2415 ± 1662.56	< 0.0001
UA (umol/L)	309.9 ± 76.07	421.1 ± 42.58	< 0.0001
TP (g/L)	72.3 ± 8.51	60.5 ± 7.83	< 0.0001
*Rats*			
FBG (mmol/L)	5.5 ± 0.6	23.6 ± 4.2	< 0.0001
CREA (umol/L)	21.4 ± 4.3	45.6 ± 9.7	< 0.0001
BUN (mmol/L)	2.5 ± 0.5	6.8 ± 0.9	< 0.0001
24hUTP (mg)	45.6 ± 6.1	320.5 ± 68.3	< 0.0001

Data presented as mean ± SD. BMI, body mass index; SBP, systolic blood pressure; DBP, diastolic blood pressure; FBG, fasting blood glucose; CREA, creatinine; CCr, creatinine clearance; 24hUTP, 24-h urinary protein quantity; UA, uric acid; TP, total protein; BUN, blood urea nitrogen

The characteristics of the rats in the current study are presented in Table1. Consistent with the results from participants, renal TNFα and IL-6 mRNA expression was augmented in DN rats (Fig. 1f, g). Moreover, Masson trichrome staining (Fig. 1h) showed that glomerular injury and fibrosis were present and more severe in DN rats. The levels of p-p65 and WNT5A in kidney tissues were also higher in DN than control rats (Fig. 1i, j; Additional file 2: Fig. 2S1a, b).

### High glucose elevated inflammatory factors levels and p-p65 expression through elevation of WNT5A levels in HGECs

**Fig. 2:**
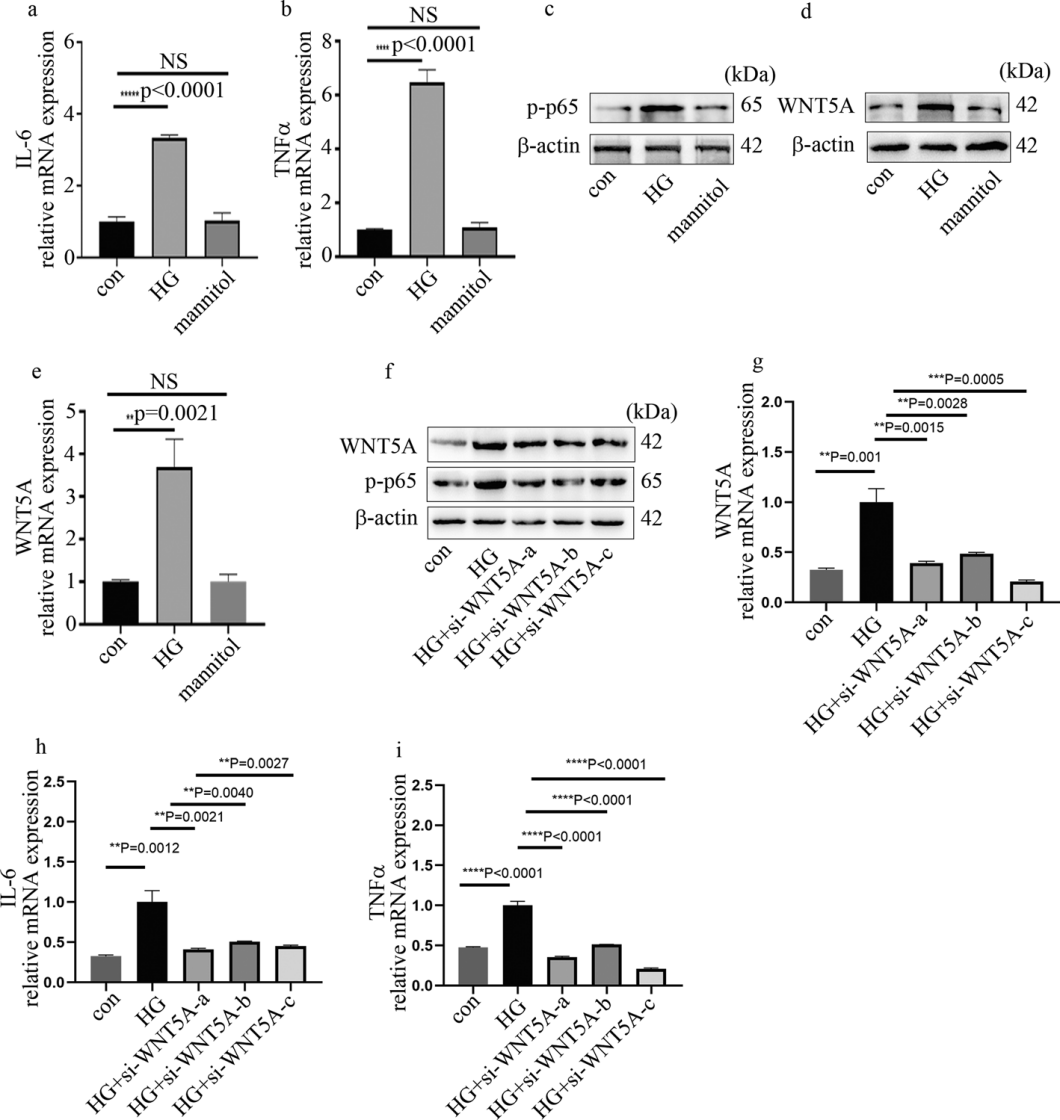
High-glucose-induced inflammatory factors and p-p65 expression was reversed by si-WNT5A. **a**, **b** mRNA expression of IL-6 and TNFα in HGECs. **c**, **d** Western blot analysis of p-p65 and WNT5A in HGECs. **e** mRNA expression of WNT5A in HGECs. **f** Western blot analysis of p-p65 and WNT5A in HGECs. **g**–**i** mRNA expression of WNT5A, IL-6, and TNFα in HGECs (*p < 0.05, **p < 0.01, ***p < 0.001, ****p < 0.0001, *n* = 5 per group)

To explore whether WNT5A regulated endothelial inflammation in glomeruli, HGECs were applied in the current study. The results showed that, compared with control HGECs, inflammatory factors (Fig. 2a, b) and p-p65 (Fig. 2c) expression were elevated in hyperglycemic HGECs. WNT5A expression in HGECs were also detected in the current study. The results showed that, compared with control HGECs, WNT5A protein (Fig. 2d) and mRNA (Fig. 2e) levels were elevated in hyperglycemic HGECs. However, mannitol was ineffective in regulation of cellular inflammatory factors or WNT5A expression in HGECs (Fig. 2a–e). To further investigate the role of WNT5A in high-glucose-induced p-p65 and inflammatory factor levels in HGECs, three independent siRNAs against WNT5A were used; their effects were confirmed by western blotting (Fig. 2f) and qPCR (Fig. 2g). The present study showed that si-WNT5A treatments inhibited high-glucose-induced p-p65 (Fig. 2f) and cellular inflammatory factors (Fig. 2h, i) levels in HGECs. Taken together, these results demonstrate that WNT5A positively regulates endothelial inflammatory factors levels in hyperglycemic HGECs.

### SETD8 was involved in high-glucose-induced p-p65 and inflammatory factors levels through modulation of WNT5A levels in HGECs

**Fig. 3:**
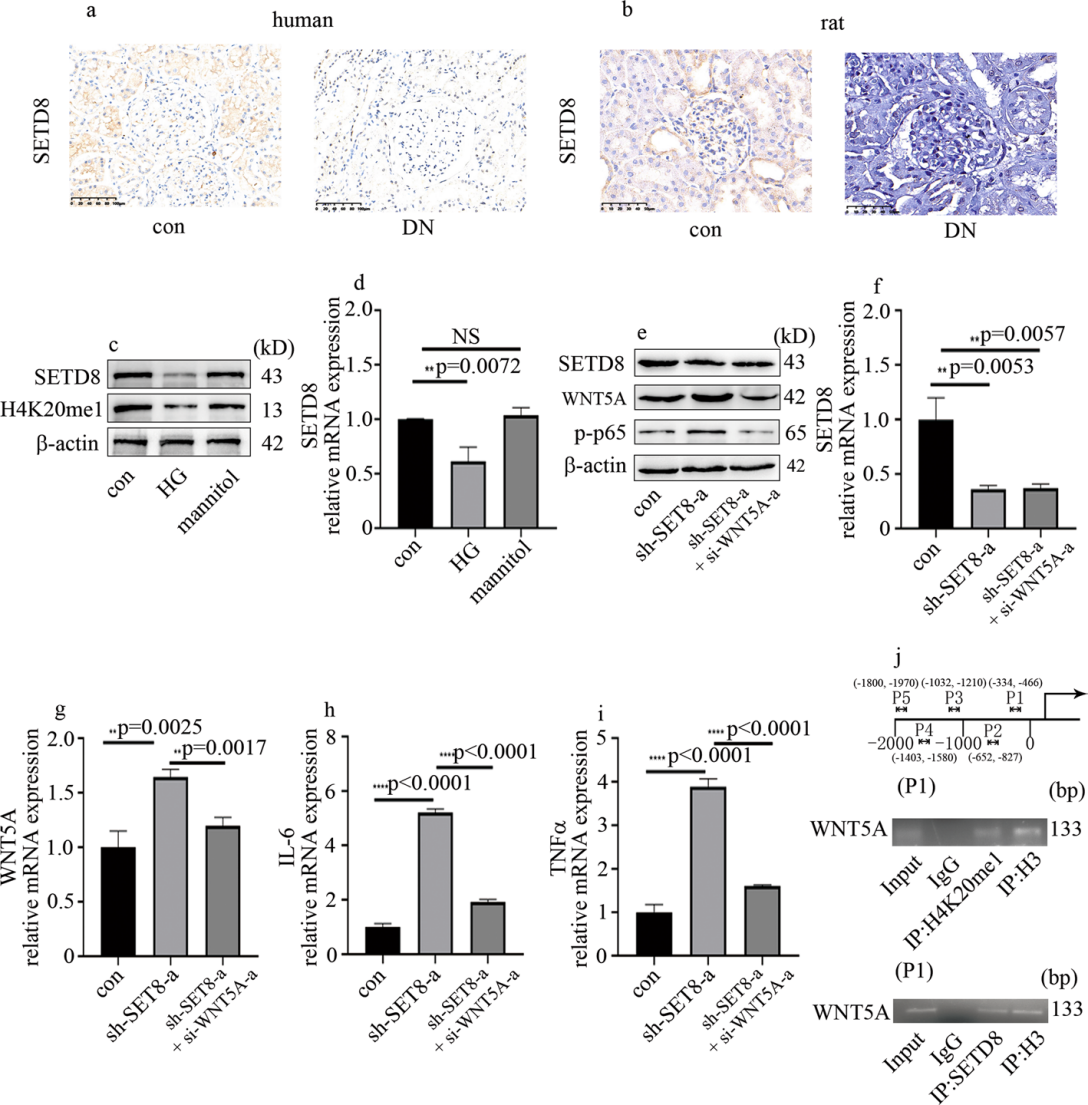
SETD8 was involved in high-glucose-induced p-p65 and inflammatory factors levels through modulation of WNT5A levels in HGECs. **a**, **b** IHC expression of SETD8 in human and rat. **c** Western blot analysis of SETD8 and H4K20mel in HGECs. **d** mRNA expression of SETD8 in HGECs. **e** Western blot analysis of SETD8, WNT5A, and p-p65 in HGECs. **f**–**i** mRNA expression of SETD8, WNT5A, IL-6, and TNFα in HGECs. **j** SETD8 and H4K20mel were enriched in the WNT5A promoter (*p < 0.05, **p < 0.01, ***p < 0.001, ****p < 0.0001, *n* = 5 per group)

Epigenetics play a crucial role in hyperglycemic vascular endothelial injury. Moreover, our previous studies illustrated that SETD8 participates in the pathogenesis of DN [19]. IHC assay indicated that SETD8 was downregulated in glomeruli of DN participants and rats (Fig. 3a, b). Consistently, the levels of SETD8 were decreased in DN rats and hyperglycemic HGECs (Fig. 3c, d; Additional file 3; Fig. S2a, b). Consistently, compared with control HGECs, the levels of H4K20me1, a downstream target of SETD8, were reduced in hyperglycemic HGECs (Fig. 3c). Moreover, mannitol was ineffective on SETD8 and H4K20me1 expression in HGECs (Fig. 3c, d). To investigate the effects of SETD8 on high-glucose-induced WNT5A levels and cellular inflammation, we employed both gain-of-function and loss-of-function experiments. The effect of SETD8 overexpression in hyperglycemic HGECs was proved by western blot (Additional file 3: Fig. S2c) and qPCR (Additional file 3: Fig. S2d). SETD8 overexpression reversed high-glucose-induced WNT5A expression (Additional file 3: Fig. S2c, e), p-p65 expression (Additional file 3: Fig. S2c), and endothelial inflammatory factors levels (Additional file 3: Fig. S2f, g) in HGECs. On the contrary, the effect of sh-SETD8 was similar to that of high-glucose treatment (Additional file 3: Fig. S2h-l). To determine whether the effects of sh-SETD8 were due to increased WNT5A expression, we inhibited expression of WNT5A through siRNA in HGECs in which SETD8 was downregulated (Fig. 3e–i). Our data indicated that WNT5A inhibition counteracts SETD8-silencing-induced p-p65 expression and endothelial inflammation in HGECs (Fig. 3e–i). These data indicate that SETD8 silencing increases p-p65 expression and endothelial inflammatory factors levels by increasing WNT5A expression. To evaluate whether WNT5A is directly targeted by SETD8, a ChIP experiment was employed to detect the genomewide distribution of SETD8 and H4K20me1 in HGECs. The data indicated that SETD8 and H4K20me1 both located in the promoter region of WNT5A (Fig. 3j).

### MZF1 interacts with SETD8

**Fig. 4:**
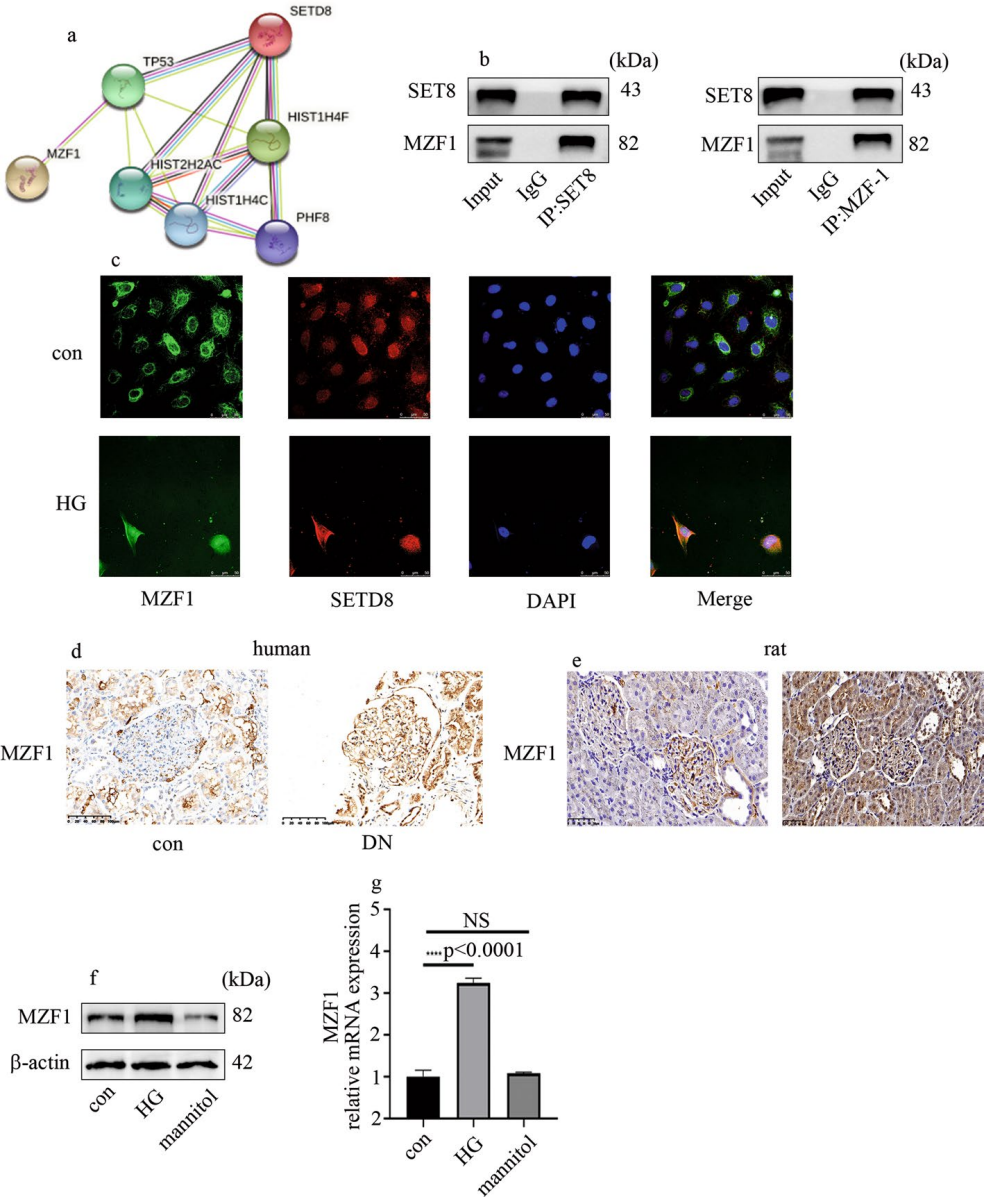
MZF1 interacts with SETD8 in HGECs. **a** Bioinformatics analysis of SETD8. **b** Association between SETD8 and MZF1 verified by CoIP. **c** Colocalization of MZF1 and SETD in HGECs. **d, e** IHC expression of MZF in human and rat. **f, g** Protein and mRNA levels of MZF1 in HGECs (*p < 0.05, **p < 0.01, ***p < 0.001, ****p < 0.0001, *n* = 5 per group)

To detect the potential mechanism of SETD8 regulating WNT5A expression in HGECs, the bioinformatics method was used to predict which transcription factor interacts with SETD8, and MZF1 was found in this study. Figure 4a shows the proteins that interacted with SETD8. The interaction between MZF1 and SETD8 in HGECs was confirmed by CoIP experiments (Fig. 4b). Immunofluorescent staining also proved the colocalization of MZF1 and SETD8 in HGECs (Fig. 4c). Moreover, compared with control HGECs, high-glucose-treatment mediated MZF1 nuclear translocation (Fig. 4c). Further, MZF1 levels were found to be elevated in DN participants and rats (Fig. 4d, e; Additional file 4: Fig. S3a, b). Consistently, compared with control HGECs, protein and mRNA levels of MZF1 were increased in hyperglycemic HGECs, with mannitol ineffective (Fig. 4f, g).

### MZF1 participated in high-glucose-induced inflammation through upregulation of WNT5A levels in HGECs

**Fig. 5:**
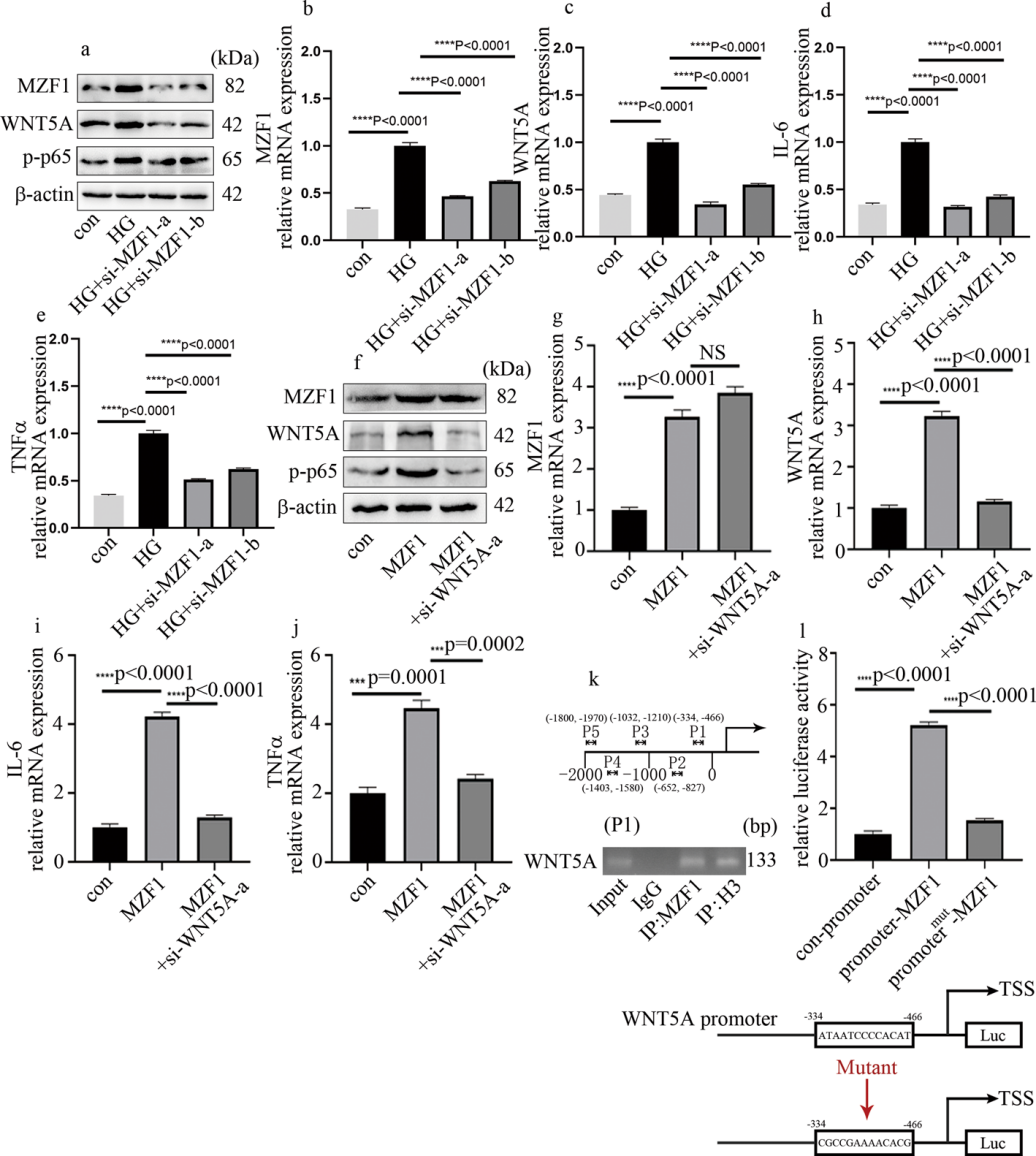
MZF1 regulates WNT5A expression and inflammatory factors in HGECs. **a** Western blot analysis of MZF1, p-p65, and WNT5A in HGECs. **b**–**e** mRNA expression of MZF1, WNT5A, IL-6, and TNFα in HGECs. **f** Western blot analysis of MZF1, p-p65, and WNT5A in HGECs. **g**–**j** mRNA expression of MZF1, WNT5A, IL-6, and TNFα in HGECs. **k** MZF1 in promoter region of WNT5A. **l** Luciferase assays of effect of MZF1 on WNT5A promoter activity (*p < 0.05, **p < 0.01, ***p < 0.001, ****p < 0.0001, *n* = 5 per group)

To prove whether MZF1 regulated WNT5A levels and inflammation in hyperglycemic HGECs, both loss-of-function and gain-of-function approaches were used. The effect of si-MZF1 is shown in Fig. 5a, b. MZF1 silencing reversed high-glucose-induced WNT5A levels (Fig. 5a, c). Moreover, si-MZF1 reversed high-glucose-induced IL-6 and TNFα levels (Fig. 5d, e) and p-p65 expression (Fig. 5a) in HGECs. Further, the impact of MZF1 upregulation was similar to that of the high-glucose treatment (Fig. 5f–j). To prove whether the impact of MZF1 upregulation was acquired by elevation of WNT5A levels, we employed si-WNT5A in MZF1-overexpressing cells. Our data indicated that WNT5A downregulation reversed MZF1-upregulation-induced p-p65 (Fig. 5f) and inflammatory factors levels (Fig. 5i, j). To validate whether WNT5A is directly targeted by MZF1, a ChIP experiment was performed to detect the genomewide distribution of MZF1 in HGECs. The results demonstrated that MZF1 located in the promoter region of WNT5A (Fig. 5k). Moreover, the binding site (−126 bp to −139 bp) was confirmed by the results of luciferase assays, which demonstrated that, when the sequence of the binding side was mutated, the positive impact of MZF1 on the luciferase reporter disappeared (Fig. 5l). Taken together, these data indicate that MZF1 upregulation elevated WNT5A levels in hyperglycemic HGECs, thus enhancing the inflammatory levels.

### MZF1 synergized with SETD8 to modulate transcriptional activity of WNT5A in HGECs

**Fig. 6:**
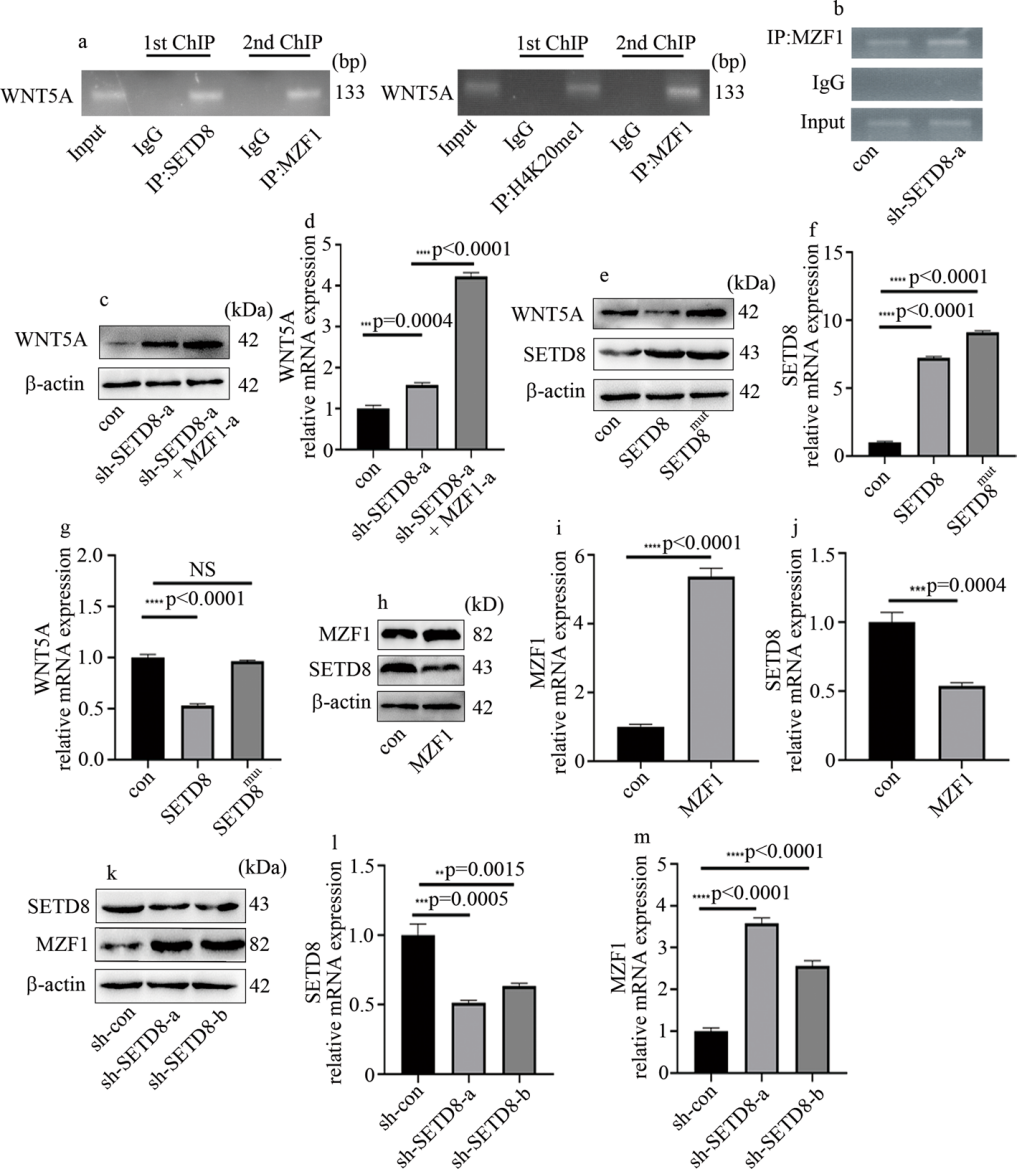
MZF1 cooperates with SETD8 to regulate WNT5A expression in HGECs. **a** Re-ChIP experiments of WNT5A. **b** Enrichment of MZF1 in WNT5A promoter region when SETD8 silenced. **c** Western blot analysis of WNT5A in HGECs. **d** mRNA expression of WNT5A in HGECs. **e**–**g** Protein and mRNA levels of SETD8 and WNT5A in HGECs. **h**–**j** Protein and mRNA levels of SETD8 and MZF1 in HGECs. **k**–**m** Protein and mRNA levels of MZF1 and SETD8 in HGECs (*p < 0.05, **p < 0.01, ***p < 0.001, ****p < 0.0001, *n* = 5 per group)

In the current study, re-ChIP results indicated that MZF1 and SETD8 located in the same WNT5A promoter region (Fig. 6a). Moreover, the enrichment of MZF1 on the WNT5A promoter region was increased when SETD8 was silenced (Fig. 6b). To further explore whether MZF1 synergized with SETD8 to regulate WNT5A expression in HGECs, we employed sh-SETD8 and overexpression of MZF1 approaches at the same time. The results showed that overexpression of MZF1 not only increased WNT5A but also enhanced the positive effect of sh-SETD8 on WNT5A expression (Fig. 6c, d). These data show that MZF1 cooperates with SETD8 to modulate WNT5A transcription in hyperglycemic HGECs. Moreover, SETD8 overexpression attenuated WNT5A expression, but the mutant SETD8^R259G^ did not affect WNT5A expression (Fig. 6e, f). These data indicate that SETD8-regulated H4K20me1 is necessary to modulate WNT5A transcription in HGECs. Moreover, mutual inhibition between SETD8 and MZF1 was determined in HGECs (Fig. 6h–m).

### SETD8 overexpression through adreno-associated virus (AVV) carriers alleviated renal injury in DN rats

**Fig. 7:**
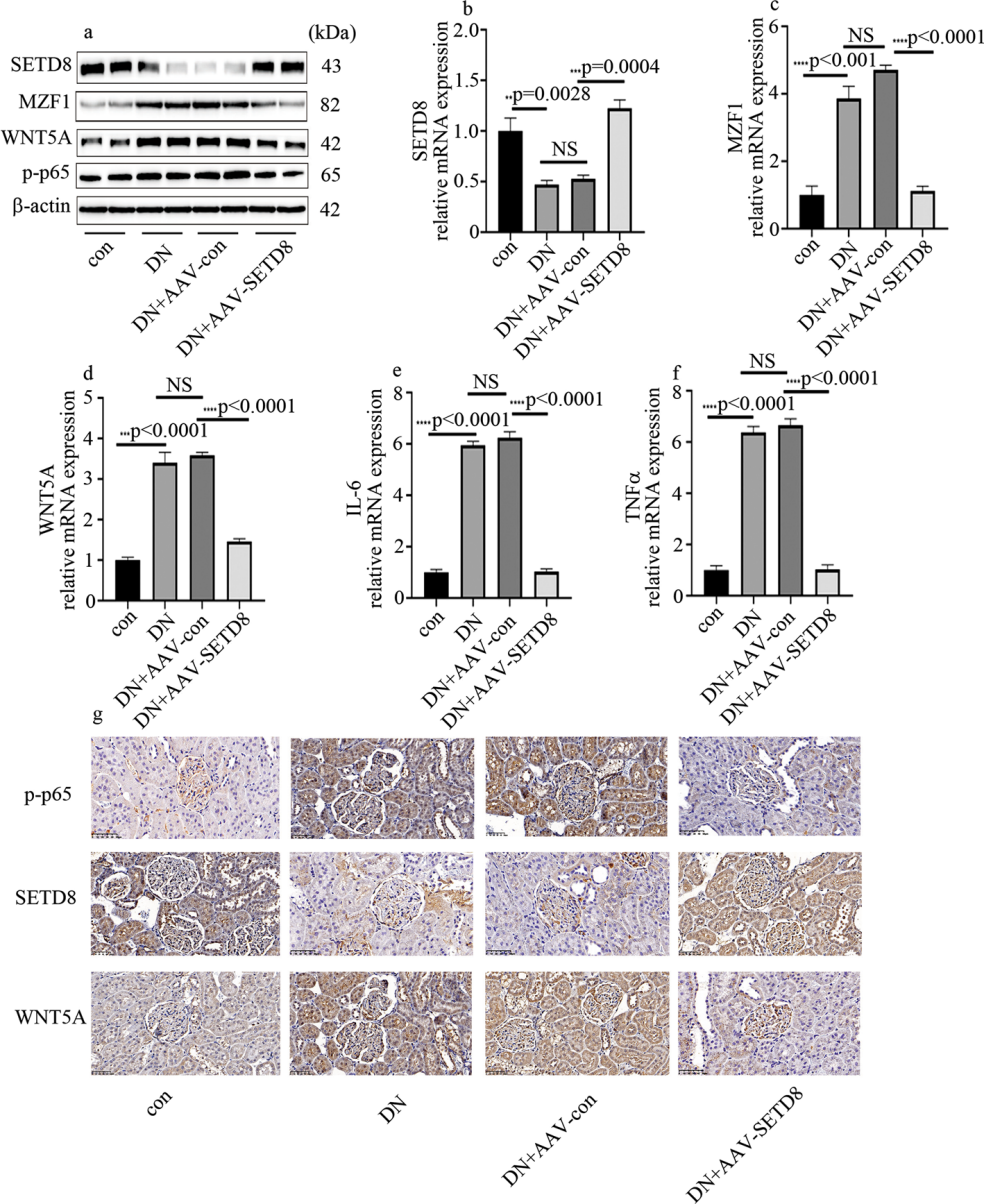
SETD8 overexpression in rats alleviated inflammation levels. **a** Western blot analysis of SETD8, MZF1, p-p65, and WNT5A in control and DN rats. **b**–**f** mRNA expression of SETD8, MZF1, WNTuo5A, IL-6, and TNFα in control and DN rats. **g** IHC expression of p-p65, SETD8, and WNT5A in control and DN rats (*p < 0.05, **p < 0.01, ***p < 0.001, ****p < 0.0001, *n* = 10 per group)

We next evaluated the potential protective effects of SETD8 overexpression on kidney injury in DN rats using a treatment protocol in which DN rats were injected with AAV-SETD8 or control vectors into the contralateral kidney at the time of Unx. The effects of SETD8 overexpression were confirmed by western blotting (Fig. 7a), qPCR (Fig. 7b), and IHC (Fig. 7g). Compared with the control group, both protein and mRNA expression levels of MZF-1, WNT5A, and p-p65 were increased significantly in the DN group (Fig. 7a–d, g). In line with this, mRNA levels of inflammatory factors were also increased in the DN group (Fig. 7e, f). SETD8 overexpression significantly decreased the expression of these factors (Fig. 7c–g) and improved the renal function of DN rats (Additional file 5: Fig. S4a–d).

## Discussion

Endothelial cells are believed to be the first responder to hyperglycemia, and endothelial dysfunction is the major pathological mechanism underlying the occurrence and development of diabetes-related vascular disease [22]. The present study reveals that high glucose participates in the regulation of endothelial inflammation levels by increasing the expression of endothelial WNT5A, and ultimately leads to the damage of glomerular endothelial cells. At the same time, high glucose increased MZF1 expression and reduced SETD8 expression, and both located on the promoter region of WNT5A. Mechanistic studies showed that MZF1 and SETD8 cooperated with each other to regulate WNT5A transcription, thus increasing levels of endothelial inflammatory factors and p-p65 expression in hyperglycemic HGECs.

Inflammatory pathways are reported to be activated in the genesis and development of DN. Proinflammatory cytokines such as IL-6 and TNFα are also involved in the pathogenesis of DN [6, 7, 23]. As inflammation continues in DN, fibroblasts will be recruited subsequently to result in renal fibrosis and injury. WNT5A was reported to regulate NF-κB activity and a series of cytokines [24]. In the current study, WNT5A expression was enhanced in glomeruli of DN patients and rats (Fig. 1). In vitro, inhibition of WNT5A through siRNA reversed high-glucose-induced p-p65 expression, thus mitigating levels of endothelial inflammatory factors in HGECs (Fig. 2). Our data may suggest that inhibiting expression of WNT5A in glomerular endothelial cells can reduce renal injury caused by hyperglycemia.

Our previous study indicated that SETD8 is involved in many pathways associated with hyperglycemia-mediated endothelial injury, such as high-glucose-induced endothelial proinflammatory enzyme and proinflammatory cytokine production, NOD-like receptor pyrin domain 3 inflammasome (NLRP3)activation, antioxidant imbalance, and endothelial adhesion molecule expression [25–27]. In the current study, SETD8 expression was reduced in DN participants, DN rats, and hyperglycemic HGECs (Fig. 3; Additional file 3: Fig. S2). The effect of SETD8 knockout was similar to that of high glucose, which could be reversed by si-WNT5A (Fig. 3). SETD8 and H4K20mel were both enriched in the promoter region of WNT5A (Fig. 3). SETD8 overexpression inhibited high-glucose-induced WNT5A expression and p-p65 expression in HGECs, thus alleviating high-glucose-induced inflammatory factors levels (Additional file 3: Fig. S2). Consistently, our in vivo study showed that SETD8 overexpression reduced WNT5A expression, p-p65 expression, and inflammatory factors levels, thus improving renal dysfunction in DN rats (Fig. 7; Additional file 4: Fig. S3). These data indicated that upregulation of SETD8 levels may be an effective treatment approach to deal with DN.

MZF1 was firstly isolated from a cDNA library made from a patient with chronic myelogenous leukemia. MZF1 has been indicated to regulate various factors in many cancers and induces migration, invasion, and in vivo metastatic potential in solid tumor cells [28]. Moreover, MZF1 is involved in the transformation of mesenchymal stem cells into cancer-associated fibroblasts [29]. In the present study, we found that MZF1 was enriched in the promoter region of WNT5A (Fig. 5). MZF1 knockout inhibited high-glucose-induced WNT5A and p-p65 expression, thus attenuating levels of inflammatory factors in HGECs (Fig. 5). Furthermore, the effect of MZF1 overexpression is similar to that of the high-glucose stimulation (Fig. 5). Further, WNT5A knockout through siRNA reversed MZF1-overexpression-induced p-p65 expression and inflammatory factors levels (Fig. 5). These data indicate that overexpression of MZF1 increases high-glucose-mediated p-p65 expression and inflammatory factors levels by upregulating expression of WNT5A, which contributes to the genesis and development of DN.

The transcriptional activity of MZF1 is regulated by epigenetic modifications [30]. The present study suggests a connection between MZF1 and SETD8 in HGECs (Fig. 4). Moreover, MZF1 and SETD8 both occupy the promoter region of WNT5A, and SETD8 knockout increased the enrichment of MZF1 on WNT5A promoter (Fig. 6). Further, MZF1 and SETD8 inhibited each other (Fig. 6) and cooperated to regulate WNT5A transcription in hyperglycemic HGECs (Fig. 6). Moreover, SETD8 overexpression inhibited WNT5A expression, whereas the mutant SETD8^R259G^ did not have this effect (Fig. 6). These results demonstrate that SETD8 regulated WNT5A expression through H4K20me1. In vivo, WNT5A, MZF1, and p-p65 expression and inflammatory factors levels were decreased by overexpression of SETD8, and the kidney function of DN rats also improved (Fig. 7).

Some limitations of our study should be considered. First, our study was only validated by HGECs, and other endothelial cells should be used to investigate further. Second, the potential mechanism of mutual inhibition between SETD8 and MZF1 is still not well known. It may be related to the duration of hyperglycemia, which deserves further study. Third, our study only used the SETD8 overexpression model of RN rats; a gene knockout model could be used further to validate our results from the opposite point. Fourth, the levels of serum inflammatory factors in renal cancer patients, who were employed as the control group in this study, may be differ from those of healthy people [31]. However, only stage I–II renal cancer patients were employed in the present study; their serum inflammatory factor levels are reported to show no marked difference compared with healthy participants, especial for IL-6 [32]. Moreover, nondiabetic renal cancer patients with normal renal function are usually used as a control group to study inflammation responses in DN [33]. In future research, healthy participants should be employed as control group to continue our study. Fifth, IL-6 and TNFα mRNA levels in tissue of participants should be provided, instead of the serum IL-6 and TNFα levels used in this study. However, serum IL-6 and TNFα have been reported to be used as predictors of kidney disease progression in diabetic nephropathy [34, 35]. Moreover, the variation tendency of levels of serum inflammatory factors in participants was similar to that of mRNA expression of inflammatory factors in in vivo and in vitro studies in the present study.

## Conclusions

The results of the current study suggest that MZF1 and WNT5A expression were increased and SETD8 expression was decreased in glomeruli of DN patients and rats. High-glucose-induced WNT5A expression is the reason for the elevation of p-p65 expression and inflammatory factors levels in hyperglycemic HGECs. SETD8 expression was decreased and MZF1 expression was increased under the stimulus of high glucose. SETD8 and MZF1 inhibit each other but cooperate to regulate WNT5A expression, p-p65 expression, and inflammatory factors levels, thus impacting the genesis and development of DN.

## Supplementary Information

**Additional file 1: Table S1.** Primers used for real-time RT-PCR analysis.

**Additional file 2: Fig. S1.** WNT5A and p-p65 levels were increased in DN rats. **a** Protein expression of WNT5A and p-p65 in renal tissue of control and DN rats. **b** mRNA expression of WNT5A in renal tissue of control and DN rats. (*p < 0.05, **p < 0.01, ***p < 0.001, ****p < 0.0001, *n* = 10 per group)

**Additional file 3: Fig. S2.** WNT5A expression and inflammatory factors levels in HGECs were regulated by SETD8. **a** Western blot analysis of SETD8 in renal tissue of control and DN rats. **b** mRNA expression of SETD8 in renal tissue of control and DN rats. **c** Protein levels of SETD8, WNT5A, and p-p65 in HGECs. **d**–**g** mRNA levels of SETD8, WNT5A, IL-6, and TNFα in HGECs. **h** Protein levels of SETD8, WNT5A, and p-p65 in HGECs. **d**–**g** mRNA levels of SETD8, WNT5A, IL-6, and TNFα in HGECs. (*p < 0.05, **p < 0.01, ***p < 0.001, ****p < 0.0001, *n* = 5 per group)

**Additional file 4: Fig. S3.** MZF1 expression were increased in DN rats. **a** Protein expression of MZF1 in renal tissue of control and DN rats. **b** mRNA expression of MZF1 in renal tissue of control and DN rats. (*p < 0.05, **p < 0.01, ***p < 0.001, ****p < 0.0001, *n* = 10 per group)

**Additional file 5: Fig. S4.** Renal function indexes were improved when SETD8 was overexpressed in DN rats. **a**–**d** Fasting blood sugar (FBS), creatinine (CREA), blood urea nitrogen (BUN), and 24-h urinary protein(24UTP) in plasma of rats

## Data Availability

The datasets used and/or analyzed during the current study are available from the corresponding author on reasonable request.

## Abbreviations

DN: Diabetic nephropathy
WNT5A: Wingless-type MMTV integration site family member 5A
SETD8: SET domain-containing protein 8
MZF1: Myeloid zinc finger 1
IL-6: Interleukin-6
TNFα: Tumor necrosis factor α
p-p65: P65 phosphorylation
