# Supplementary material for: SETD8 cooperates with MZF1 to participate in hyperglycemia-induced endothelial inflammation via elevation of WNT5A levels in diabetic nephropathy
Source: Cell Mol Biol Lett. 2022 Mar 26;27:30. doi: 10.1186/s11658-022-00328-6 (PMC8962284; doi:10.1186/s11658-022-00328-6)
Supplement: Supplementary file 1 — Additional file 1: Table S1. Primers used for real-time RT-PCR analysis. [file 11658_2022_328_MOESM1_ESM.docx]

| species | RNA sequence |
| --- | --- |
| Human  β-actin  SETD8  MZF-1  WNT5A  IL6  TNFα    Rat  β-actin  SETD8  MZF-1  WNT5A  TNFα    IL-6 | F 5’- AGCGAGCATCCCCCAAAGTT -3’  R 5’- GGGCACGAAGGCTCATCATT -3’  F 5’- TCCAGCAATCCTCCTCCTTCCTC -3’  R 5’- CCAGCCTAAGCAACAGATCCAGA -3’  F 5’- GGCTGCTGCCCTAGTAGATG -3’  R 5’- TCTGGAGTTGGAGGCTCAGT -3’  F 5’- GATCATCCTCCCTTTCGCCC -3’  R 5’- CCAACCACAGACATCGTCCA-3’  F 5’- ACTCACCTCTTCAGAACGAATTG -3’  R 5’- CCATCTTTGGAAGGTTCAGGTTG -3’  F 5’- ATGAGCACTGAAAGCATGATC-3’  R 5’- TCACAGGGCAATGATCCCAAAGTAGACCTGCCC-3’  F 5’- CTTCCAGCCTTCCTTCCTGG -3’  R 5’- GAGCCACCAATCCACACAGA -3’  F 5’- GCAGGAAGAGAACTCCGTCG -3’  R 5’- AGAATCACATGACGGGGGTG -3’  F 5’- TCCTAGGTGCACTGCCTTCT -3’  R 5’- ATAGGACCTCCTTGCCCTGT -3’  F 5’- AAAACGGTCAGTGTTGGCCT-3’  R 5’- TTAGAGCTGTGCCTCCATGC-3’  F 5’- GAAACACACGAGACGCTGAA-3’  R 5’-AAGCTCTGAGGGAAGGAAGG--3  F 5’- AGTTGCCTTCTTGGGACTGA-3’  R 5’- CAGAATTGCCATTGCACAAC-3 |

Supplementary Table 1 Primers used for real-time RT-PCR analysis.
