# Supplementary figures and images for: SETD8 cooperates with MZF1 to participate in hyperglycemia-induced endothelial inflammation via elevation of WNT5A levels in diabetic nephropathy
Source: Cell Mol Biol Lett. 2022 Mar 26;27:30. doi: 10.1186/s11658-022-00328-6 (PMC8962284; doi:10.1186/s11658-022-00328-6)

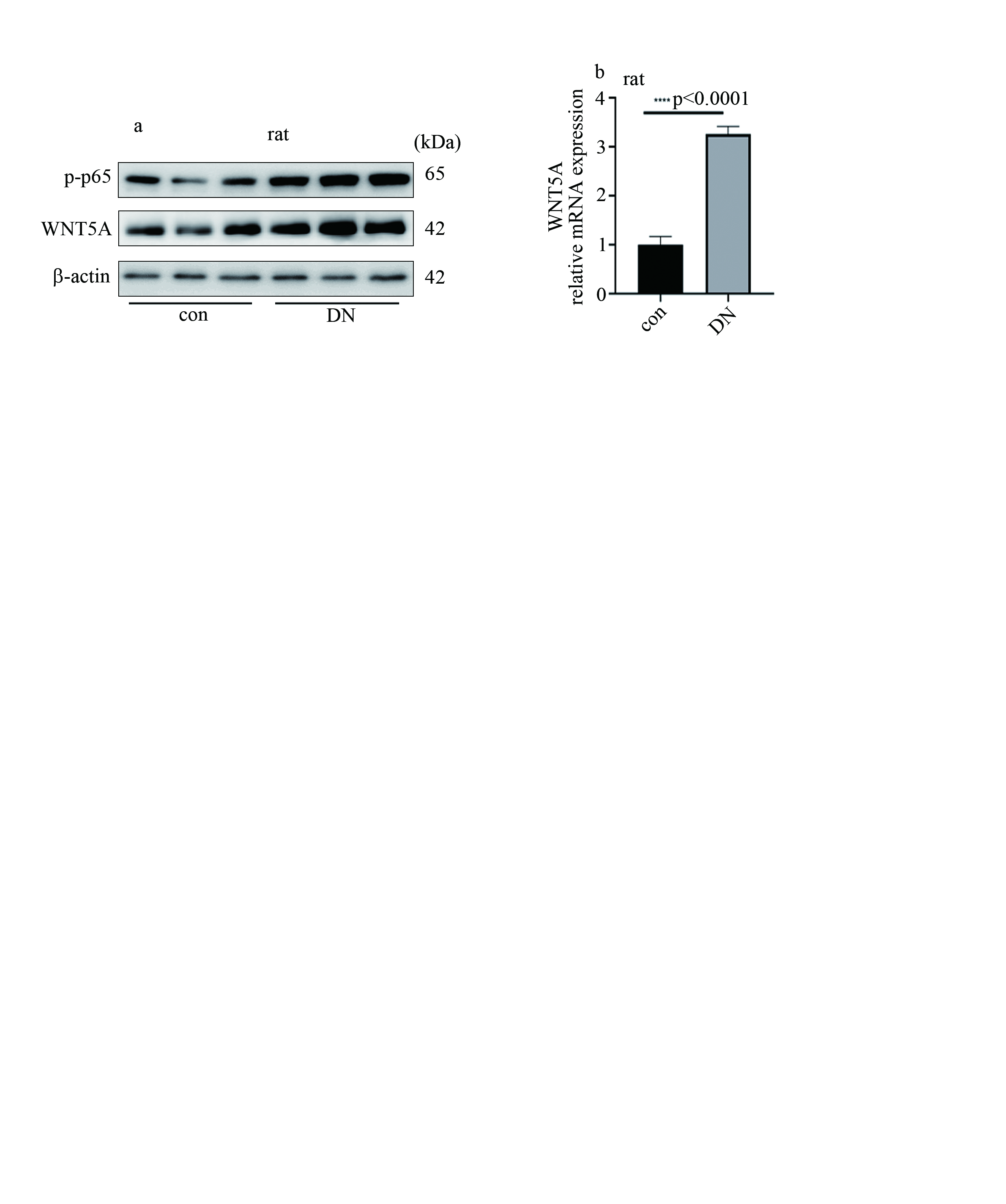

Supplement: Supplementary file 2 — Additional file 2: Fig. S1. WNT5A and p-p65 levels were increased in DN rats. a Protein expression of WNT5A and p-p65 in renal tissue of control and DN rats. b mRNA expression of WNT5A in renal tissue of control and DN rats. (*p < 0.05, **p < 0.01, ***p < 0.001, ****p < 0.0001, n = 10 per group) [file 11658_2022_328_MOESM2_ESM.tif]

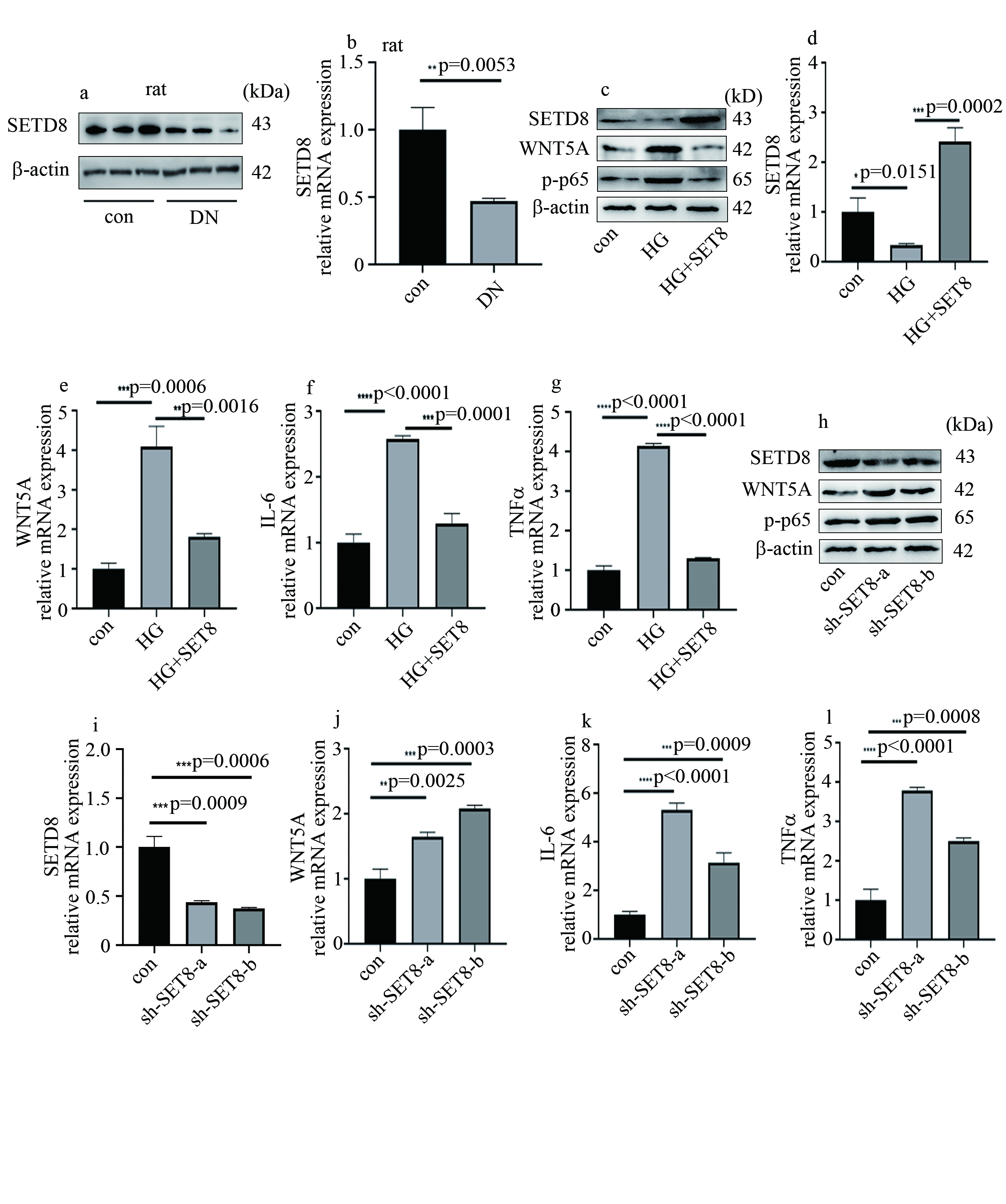

Supplement: Supplementary file 3 — Additional file 3: Fig. S2. WNT5A expression and inflammatory factors levels in HGECs were regulated by SETD8. a Western blot analysis of SETD8 in renal tissue of control and DN rats. b mRNA expression of SETD8 in renal tissue of control and DN rats. c Protein levels of SETD8, WNT5A, and p-p65 in HGECs. d–g mRNA levels of SETD8, WNT5A, IL-6, and TNFα in HGECs. h Protein levels of SETD8, WNT5A, and p-p65 in HGECs. d–g mRNA levels of SETD8, WNT5A, IL-6, and TNFα in HGECs. (*p < 0.05, **p < 0.01, ***p < 0.001, ****p < 0.0001, n = 5 per group) [file 11658_2022_328_MOESM3_ESM.tif]

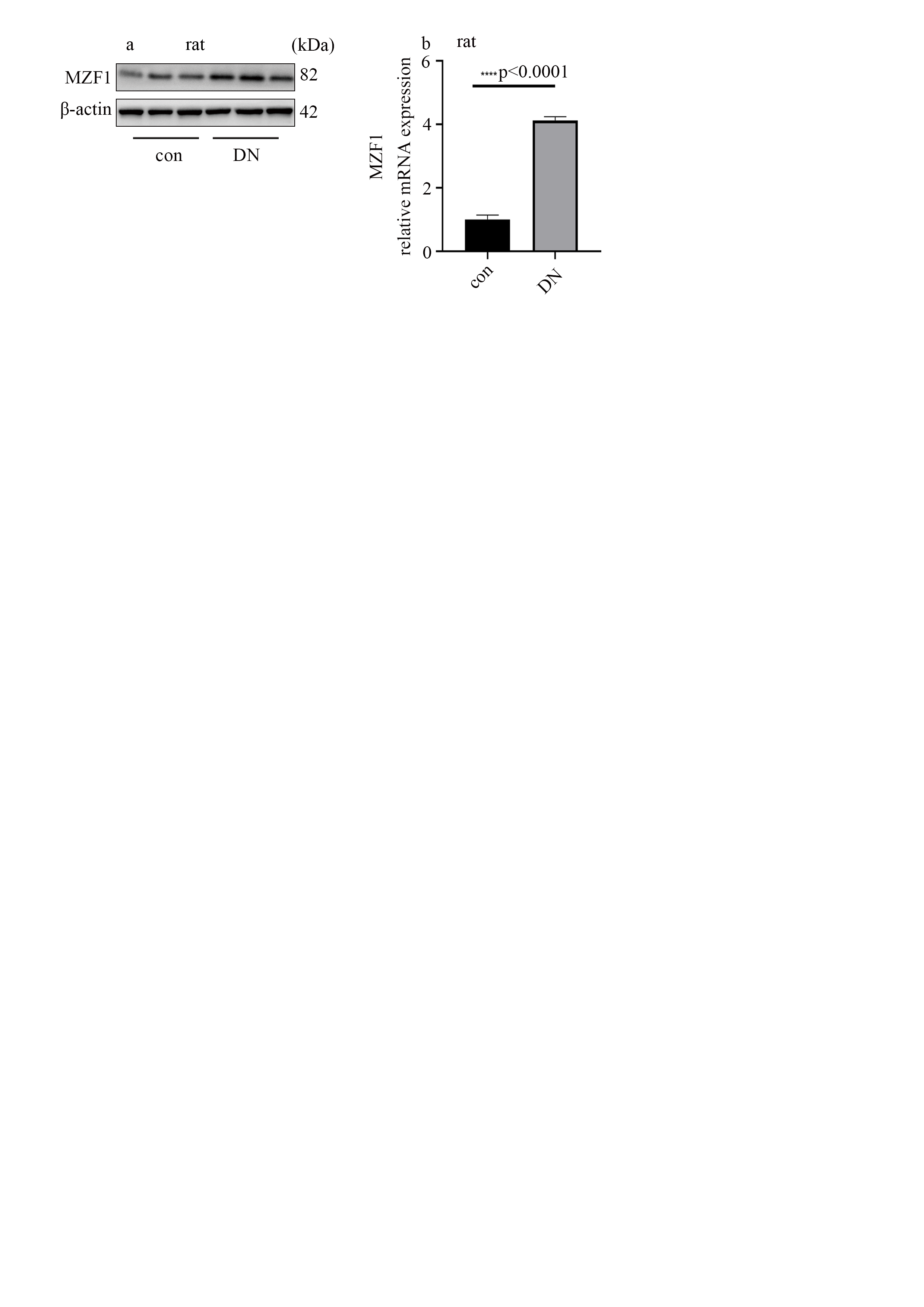

Supplement: Supplementary file 4 — Additional file 4: Fig. S3. MZF1 expression were increased in DN rats. a Protein expression of MZF1 in renal tissue of control and DN rats. b mRNA expression of MZF1 in renal tissue of control and DN rats. (*p < 0.05, **p < 0.01, ***p < 0.001, ****p < 0.0001, n = 10 per group) [file 11658_2022_328_MOESM4_ESM.tif]

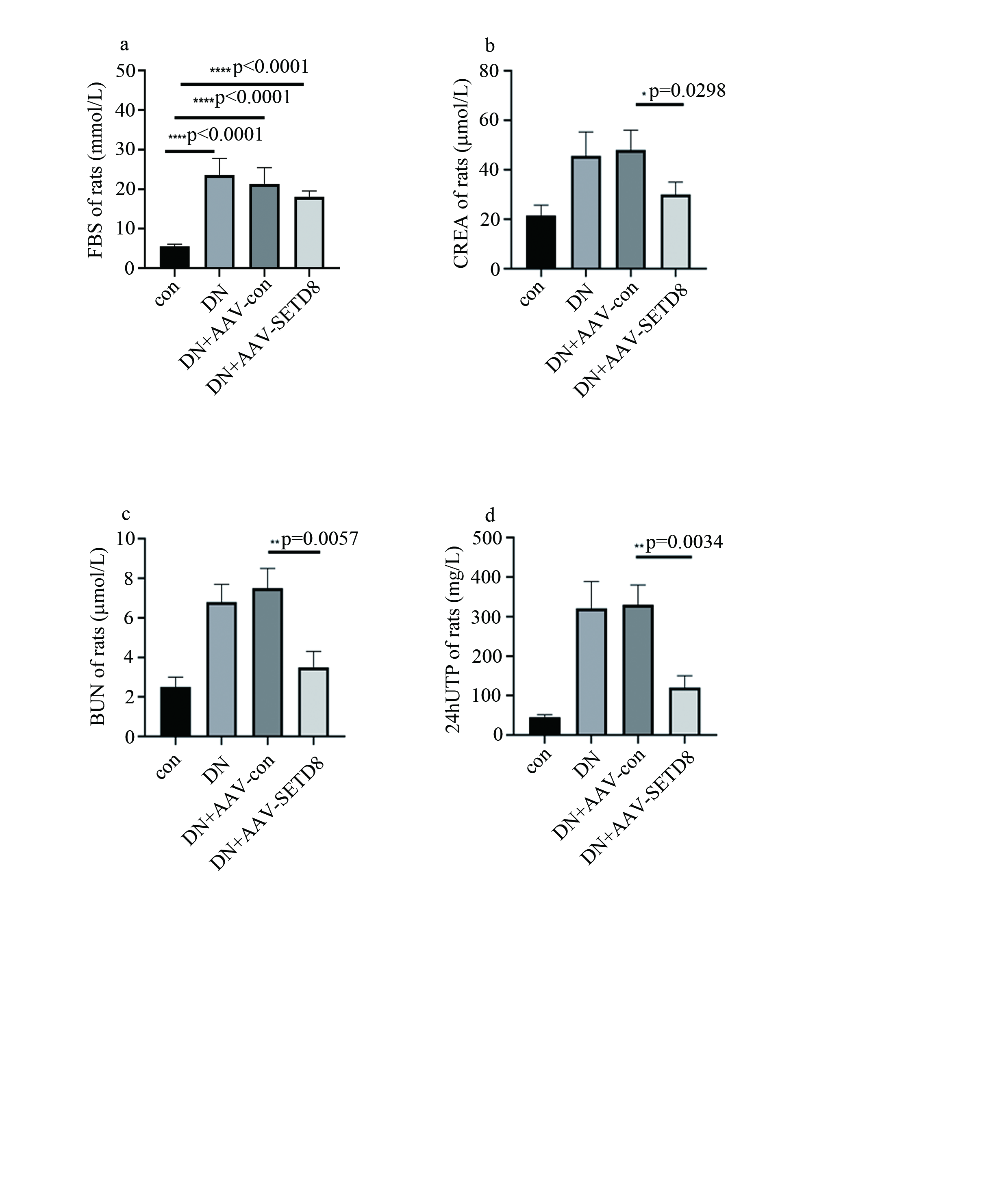

Supplement: Supplementary file 5 — Additional file 5: Fig. S4. Renal function indexes were improved when SETD8 was overexpressed in DN rats. a–d Fasting blood sugar (FBS), creatinine (CREA), blood urea nitrogen (BUN), and 24-h urinary protein(24UTP) in plasma of rats [file 11658_2022_328_MOESM5_ESM.tif]
